# Field cycling imaging to characterise breast cancer at low and ultra-low magnetic fields below 0.2 T

**DOI:** 10.1038/s43856-024-00644-2

**Published:** 2024-10-30

**Authors:** Vasiliki Mallikourti, P. James Ross, Oliver Maier, Katie Hanna, Ehab Husain, Gareth R. Davies, David J. Lurie, Gerald Lip, Hana Lahrech, Yazan Masannat, Lionel M. Broche

**Affiliations:** 1 Aberdeen Biomedical Imaging Centre, University of Aberdeen, Aberdeen, UK; 2 Institute of Biomedical Imaging, Graz University of Technology, Graz, Austria; 3 Institute of Medical Sciences, University of Aberdeen, Aberdeen, UK; 4 Breast Unit, Aberdeen Royal Infirmary, Aberdeen, UK; 5 University Grenoble Alpes, Inserm U1205, BrainTech Lab, Grenoble, France; 6 School of Medicine, Medical Sciences and Nutrition, University of Aberdeen, Aberdeen, UK

**Keywords:** Diagnostic markers, Diagnostic markers, Breast cancer, Biomarkers

## Abstract

**Background:**

This prospective feasibility study explores Field-Cycling Imaging (FCI), a new MRI technology that measures the longitudinal relaxation time across a range of low magnetic field strengths, providing additional information about the molecular properties of tissues. This study aims to assess the performance of FCI and investigate new quantitative biomarkers at low fields within the context of breast cancer.

**Methods:**

We conducted a study involving 9 people living with breast cancer (10 tumours in total, mean age, 54 ± 10 years). FCI images were obtained at four magnetic field strengths (2.3 mT to 200 mT). FCI images were processed to generate T1 maps and 1/T1 dispersion profiles from regions of tumour, normal adipose tissue, and glandular tissue. The dispersion profiles were subsequently fitted using a power law model. Statistical analysis focused on comparing potential FCI biomarkers using a Mann-Whitney U or Wilcoxon signed rank test.

**Results:**

We show that low magnetic fields clearly differentiate tumours from adipose and glandular tissues without contrast agents, particularly at 22 mT (1/T_1_, median [IQR]: 6.8 [3.9–7.8] s^−1^ vs 9.1 [8.9–10.2] s^−1^ vs 8.1 [6.2–9.2] s^−1^, *P* < 0.01), where the tumour-to-background contrast ratio was highest (62%). Additionally, 1/T_1_ dispersion indicated a potential to discriminate invasive from non-invasive cancers (median [IQR]: 0.05 [0.03–0.09] vs 0.19 [0.09–0.26], *P* = 0.038).

**Conclusions:**

To the best of our knowledge, we described the first application of in vivo FCI in breast cancer, demonstrating relevant biomarkers that could complement diagnosis of current imaging modalities, non-invasively and without contrast agents.

## Plain Language Summary

Field cycling imaging (FCI) is a new medical imaging technique that uses low and variable magnetic fields to provide information on tissue properties that cannot be obtained by other medical imaging technologies. This study aimed at finding new types of signals from FCI images to better detect and characterise breast cancer. We acquired FCI images from people living with breast cancer at four different field strengths to demonstrate that low magnetic fields enable the detection of breast cancers without the need for potentially hazardous contrast agents unlike MRI systems. Additionally, we showed that the FCI technology has the potential to provide insights into tumour invasiveness, which is not visible with current imaging modalities. These findings suggest that FCI could complement existing clinical imaging techniques to improve the diagnosis of breast cancer.

## Abstract (web summary)

Mallikourti et al. assess whether breast cancer can be detected at low magnetic fields ranging from 2.3 mT to 200 mT using endogenous T_1_ contrast, thereby eliminating the need for contrast agents. They show this method of field cycling imaging can differentiate invasive from non-invasive breast tumours in people with breast cancer.

## Introduction

Medical imaging is essential to assist physicians in management, diagnosis, follow-up, screening and in therapeutic decision-making of people living with breast cancer. Most exploit the properties of penetrative electromagnetic radiation, with different modalities associated with different energy levels: X-rays for computed tomography (SPECT-CT) and mammograms (MG), gamma rays for single-photon emission computerised tomography, positron emission tomography (PET), radiofrequency (RF) waves coupled to a magnetic field for magnetic resonance imaging (MRI) and sound waves in ultrasound (US). While each one has its specific use, MRI benefits from the good tissue penetration of RF waves and does not affect the integrity of cells or biomolecules, allowing visualisation of the whole organ non-invasively. MR-based techniques therefore have a high potential for clinics and offer many sources of anatomical and functional tissue contrast mechanisms^1^, especially when combined with contrast agents, although it is not a widespread imaging choice for the breast due to its high examination cost and limited accessibility.

Low-field MRI systems are showing a renewed interest as they offer solutions for more accessible devices. Low magnetic fields have been known for a long time to offer excellent endogenous T_1_ contrast between tissues^2–4^, but very little is known about these contrast mechanisms below 0.2 T. This is largely due to the difficulty to obtain clinically usable images using magnetic fields in the mT range and below, where SNR strongly limits the technical possibilities. Yet, many technologies are emerging that aim precisely to explore that range, using various approaches^5^.

Our approach is to explore these field regimes by using the technology of Fast-Field-Cycling Nuclear Magnetic Resonance (FFC-NMR). FFC-NMR is a long-established technique that employs rapidly varying magnetic fields with the aim to increase signal sensitivity by polarising the spin magnetisation at the highest available magnetic field strength (the polarisation field, B_0_^P^) before exploring interactions at a lower magnetic field (the evolution field, B_0_^E^). NMR signals are then measured by returning to a relatively high field (the detection field, B_0_^D^) where the Larmor frequency corresponds to that of the instrument’s RF coil. This allows measuring the longitudinal relaxation times T_1_ of proton spins over a large range of low magnetic fields using the same device, non-invasively. The output is a curve known as the T_1_ Nuclear Magnetic Resonance Dispersion (NMRD) profile, which shows the field-dependence of the longitudinal relaxation T_1_ (or more often the longitudinal relaxation rate R_1_ = 1/T_1_).

Crucially, NMRD profiles report T_1_ relaxation of water and are therefore quantitative informers of the molecular dynamics of water, but also of their neighbouring biomolecules such as proteins and lipids, spanning a time range that encompasses a large variety of water motions. It informs on the translational and rotational movements of their chemical groups, at the micro and nano-scales^6–10^. In particular, cellular transmembrane water exchange mechanisms affects T_1_ at low and ultra-low magnetic fields and have been demonstrated to be a hallmark of cancer aggressiveness in breast cancer cell lines^11^ and in invasion/migration in brain cancer tissues^11,12^. Furthermore, T_1_ at low and ultra-low magnetic fields was found to correlate with hypoxia, H_2_O_2_ oxidative stress, and to the expression of aquaporins (water channel proteins that facilitate transmembrane water transport)^12^. All these results from pioneering works show potential applications of NMR at low and ultra-low magnetic fields in cancer^13^, as well as ex vivo and in vivo results demonstrating physiological^11^ and pathophysiological mechanisms^12,14^ that affect relaxation in cancer processes, indicating a high potential for FFC-NMR as a relevant technology for diagnostic and therapy follow-up, in particular in oncology.

Interestingly, NMRD profiles of living tissues may also exhibit a series of features called quadrupolar (QP) peaks in the R_1_ = 1/T_1_ profile (or dips if one observes the T_1_ profile), visible around 65 mT, which are due to the cross-relaxation between water protons (^1^H) and the N-terminal nitrogen (^14^N) of slow-moving proteins^15,16^. QP cross-relaxations provide subtle information about protein dynamics and aggregation, and their changes can reflect some pathophysiological mechanisms such as seen in cartilage^17,18^ or blood serum^19^, and more interestingly in cancer such as in sarcoma^20^, breast^21^ and brain^12^.

Commercial FFC-NMR systems are available but can only analyse small specimens^22^, or small animals with limited localisation^11^. Our research group has built a new prototype imager called Field-Cycling Imaging (FCI, formerly FFC-MRI), which is derived from MRI but exploits FFC-NMR to access T_1_ contrast mechanisms over a very broad magnetic field strength from 20 μT to 200 mT, corresponding to proton Larmor frequency spectrum, from 850 Hz to 8.5 MHz. In addition to use rapidly switched magnets, FCI technology includes magnetic field gradients, corrective shims, and low-frequency RF coils with dedicated pulse sequences to produce images with new contrasts derived from NMRD profiles. Each image voxel therefore informs on the molecular dynamics of tissues and organs, with imaging capability over the entire body ^23^.

The standard procedure for breast cancer imaging includes MG, that can be supplemented by US scans. Both have limitations: MG is influenced by breast density while US is characterised by high false-positive rates^24,25^. MRI is used in selected cases, especially in lobular pathology, mammographically occult lesions, dense breast tissue, screening for high-risk persons/gene mutation carriers, and monitoring of treatment in Neoadjuvant Chemotherapy (NACT) while PET scans can assess the spread of cancer cells. Hybrid technologies such as PET/MRI or PET/CT systems^26^ have been proposed to improve cancer detection and target specific cell functions by using suitable radiopharmaceutical contrast agent, while contrast-enhanced mammography (CEM), which uses iodinated contrast materials, and contrast-enhanced MRI, with gadolinium-based contrast agents, locate areas with high angiogenesis and assess vessel permeability ^27^, both being correlated to cancer malignancy.

Some types of breast pathologies remain difficult to detect on MRI images and, even if detected, MRI may overestimate the extent of the disease^28^. The use of paramagnetic contrast agents is also required for MRI breast scans and there has been concern about gadolinium deposition in tissues^29^. Nevertheless, triple assessment remains the mainstay in assessing people with breast complaints, and is a combination of clinical assessment, imaging and histology diagnosis from biopsy ^30^.

In this work we study for the first time people living with breast cancer using field-cycling imaging, to the best of our knowledge. The aims of this study were (i) to investigate the potential contribution of FCI to the standard imaging in breast cancer diagnosis, and (ii) to identify quantitative FCI and low-field biomarkers related to different types of breast cancer pathology. We compare our results with existing studies at low and ultra-low fields using both ex vivo^21^ and in vivo preclinical breast cancer models^11^. We demonstrate the detection of breast cancer at low field strengths using endogenous T_1_ contrast, eliminating the need for contrast agents. We also highlight the potential of FCI to generate relevant biomarkers that could provide new diagnostic information associated with breast tumour invasiveness.

## Methods

### Cohort

This prospective feasibility study was approved by the North of Scotland Research Ethics committee (study reference 19/NS/0064), NHS Grampian and all participants gave written informed consent. This study is recorded on www.researchregistry.com under UIN number 4875.

**Fig. 1:**
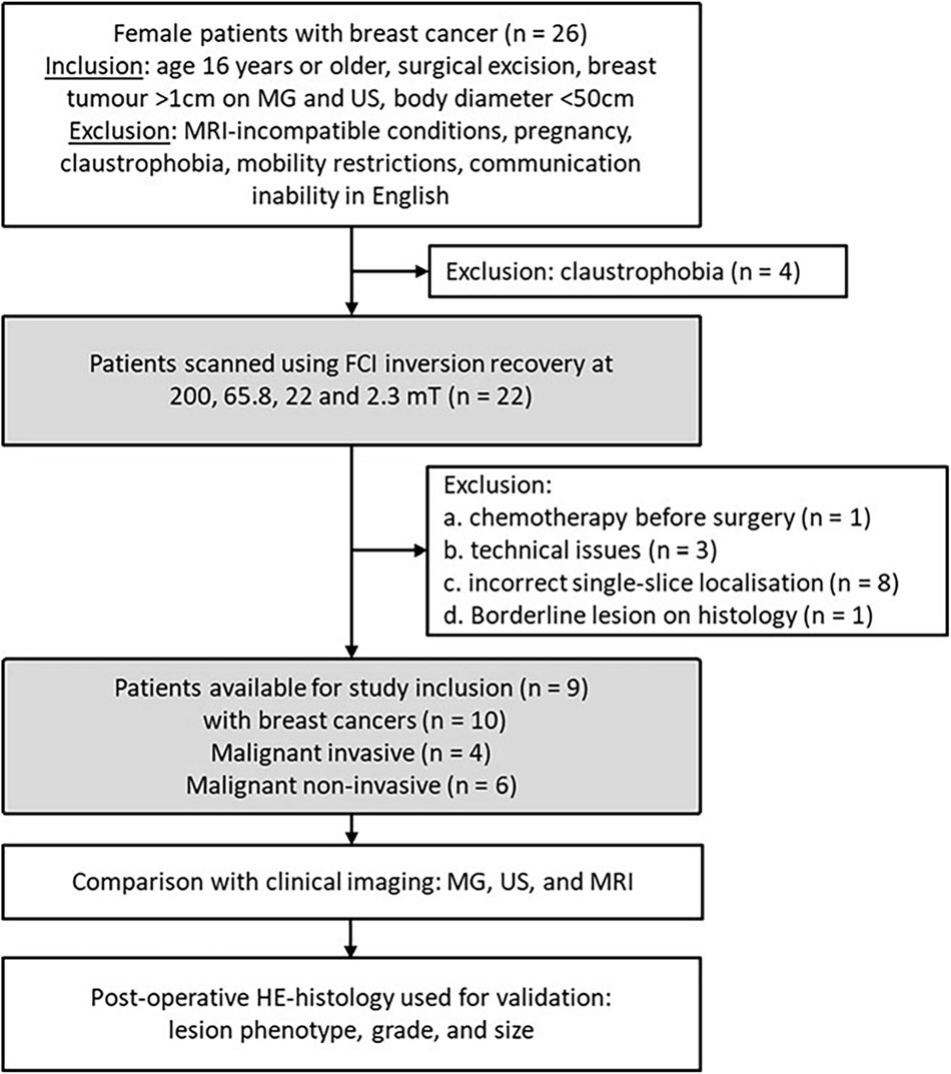
Flow diagram of the clinical protocol of volunteers undergoing FCI. This shows the criteria of inclusion and exclusion, the volunteers groups related to the cancer nature, the imaging modalities, and the standard post-operative histology analysis. (MG: Mammography, US: Ultrasounds, HE: Hematoxylin and Eosin, FCI: Field-Cycling Imaging).

Women who presented with breast cancer on conventional imaging and were treated with surgical excision were consecutively recruited from January 2019 to March 2020 and from April 2021 to March 2022 by the Breast Unit at Aberdeen Royal Infirmary. All were enroled according to the following study criteria: Inclusion criteria: (a) age 16 years or older, (b) lesion size of 1 cm or above seen on imaging, (c) suitable to fit inside the 50 cm diameter of the scanner, and (d) able to give fully informed consent. Exclusion criteria: (a) contraindications to MRI examinations, (b) pregnancy, (c) claustrophobia, (d) restrictions to mobility that would prevent the correct position in the scanner, and (d) unable to communicate in English (Fig. 1).

Twenty-six women with breast cancer were recruited in the study. Of these, 7 did not complete the scan due to claustrophobia (*n* = 4) or technical issues (*n* = 3), one volunteer was excluded because of having chemotherapy before the scan and in 8 volunteers the breast lesion was small and had not been correctly localised within the single-slice FCI scan. Ten volunteers were successfully examined by FCI. One of these was excluded because the histology report at excision showed a borderline phyllodes lesion. Finally, 9 volunteers (mean age, 54 years ± 10, age range, 41–70 years, from P1 to P9) with 10 breast cancers were included in the analysis (Table 1).

**Table 1:** Demographics of the study volunteers and tumour size as defined by each imaging modality

ID	Tumour type	Tumour Grade	Invasive	BI-RADs	Tumour size measurement^a^			
					HE Hist. (mm)	MG (mm)	US (mm)	MRI (mm)
P1	DCIS	High	No	C	51	80	NAD	-
P2	ILC	2	Yes	A	19	24	17	17
P2	DCIS	High	No		43	NAD	NAD	NAD
P3	IDC/DCIS	3/High	Yes	B	29	25	24	-
P4	PDCIS/EPC	Inter-mediate	No	C	25	41	28	-
P5	ADCIS	High	No	B	59	25	NAD/ 11	55
P6	IPLC/ILC/DCIS	2/High	Yes	A	52	20	20	64
P7	DCIS	High	No	B	36	26	NAD	42
P8	DCIS	High	No	B	32	22	12	-
P9	IDC	3	Yes	B	36	33	25	-

^a^The longest tumour size is reported.

*ADCIS* apocrine ductal carcinoma in situ, *DCIS* ductal carcinoma in situ, *EPC* Encysted papillary carcinoma, *IDC* invasive ductal carcinoma, *ILC* invasive lobular carcinoma, *IPLC* pleomorphic invasive lobular carcinoma, *PDCIS* papillary ductal carcinoma in situ, *Bord* borderline, *BI-RADs* Breast Imaging-Reporting and Data System used to define mammographic density, *MG* mammography, *NAD* no abnormality detected, *US* ultrasound.

P2 presented two distinct lesions at histology (invasive lobular carcinoma (ILC) core surrounded by a ductal carcinoma in situ (DCIS) peripheral lesion), each was treated separately for the analysis. All volunteers had a clinical MG (Hologic Selenia Dmensions, Bedford, Massachusetts) and US (Philips Affinit 70, Netherlands) and four volunteers (P2, P5, P6 and P7) had a clinically indicated 1.5 T MRI scan (Siemens 1.5 T Avanto, Erlangen, Germany) using T_2_-FLAIR, diffusion-weighted imaging, pre-post dynamic contrast-enhanced and T_1_-weighted contrast-enhanced sequences (Gadovist 1.0 mmol, Bayer, 0.1 ml/kg up to max 10 ml, 3 ml/s flow rate). Routine Hematoxylin Eosin (HE) histology was conducted at the excised lesion after surgery as per standard practice to provide lesion phenotype, grade, and size as reported in Table 1.

Breast density was scored in BI-RADs^31^ (Breast Imaging Reporting and Data System) categories A (almost entirely fatty), B (scattered areas of fibroglandular density), C (heterogeneously dense) and D (extremely dense) by an experienced consultant breast radiologist (co-author GL) (Table 1).

### FCI acquisition

**Fig. 2:**
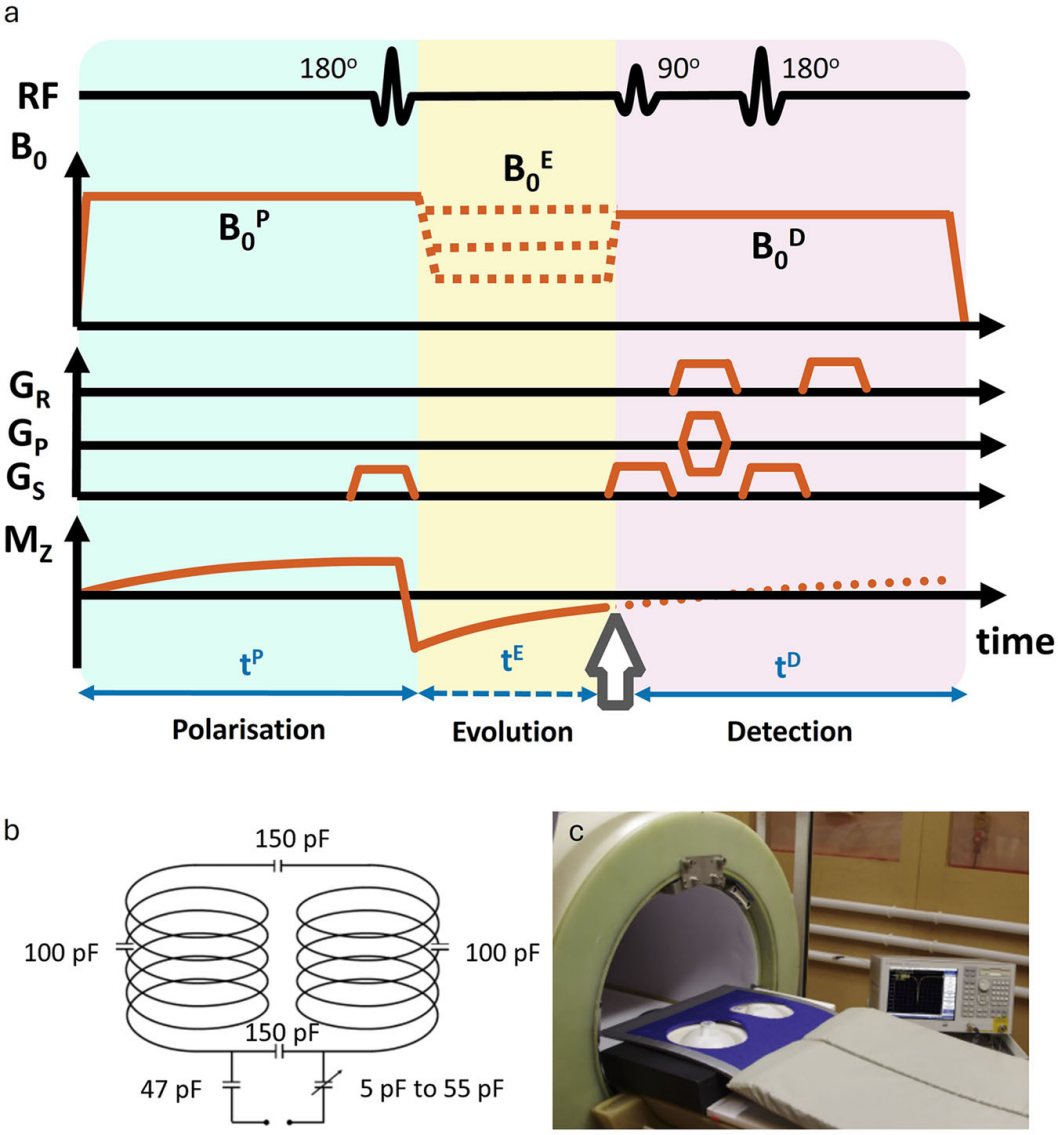
Description of the FCI hardware and pulse sequence. **a** Diagram of FCI sequence combining the inversion recovery spin echo with typical gradient echo acquisition using magnetic gradients G_R_, G_P_ and G_S_ for, read, phase and slice encoding, respectively. Mz is the longitudinal magnetisation and its polarisation is done at the highest field (here at 0.2 T during 300 ms). Signal detection is also performed at a higher field, here at 193 mT, where the Larmor proton frequency corresponds to the RF coil frequency. During acquisitions, the magnetic field is switched rapidly between the polarisation (B_0_^P^), evolution (B_0_^E^) and detection (B_0_^D^) stages to ensure good SNR. Measurements are performed with varying B_0_^E^ (illustrated by orange dashed lines) and the whole process is repeated with different evolution times (t^E^) to measure T_1_ relaxation (illustrated by blue dashed line). At the end we obtain the T_1_- NMRD profiles (T_1_ versus B_0_^E^). **b** Schematic of our home-made bilateral breast coil circuit, with a resonance frequency at 8.2 MHz for signal detection at 193 mT. The coil design was selected to provide a simple and robust transmit/receive coil with good field uniformity and as much volume as could be provided in the limitations imposed by the bore/couch rail geometry. The selected capacitor distribution was to prevent excessive voltage between any sections of the coil and to ensure that the path of the RF pulse in the coil never exceeded 1/10th of a wavelength. The loaded quality factor of the RF coil was 120, demonstrating its good performance. **c** Picture of the FCI prototype scanner used, with the breast coil in place and loaded with two phantom bottles.

Data were acquired using our FCI scanner^23^. FCI image acquisition involves three steps: polarising at a high magnetic field (polarisation field), free evolution at a lower magnetic field (evolution field), and signal acquisition at a fixed, high detection field (Fig. 2a). These steps are repeated until the k-space is fully acquired. A dedicated RF coil was designed to image both breasts simultaneously (Fig. 2b, c) at 193 mT (8.2 MHz)^32^.

The FCI scans were performed before surgery with volunteers in the prone position, using an inversion recovery spin echo sequence with an echo time of 16 ms and repetition time of 2000 ms. Imaging parameters were adapted to each volunteer, with the FOV set between 300 to 500 mm, an image size of 64 × 128, and in-plane spatial resolution of 2 to 4 mm. Due to technical limitations of our prototype FCI scanner, acquisitions were conducted on a single axial slice with a thickness of 10 mm positioned at the level of the lesion. No signal averaging or contrast agents were used.

**Fig. 3:**
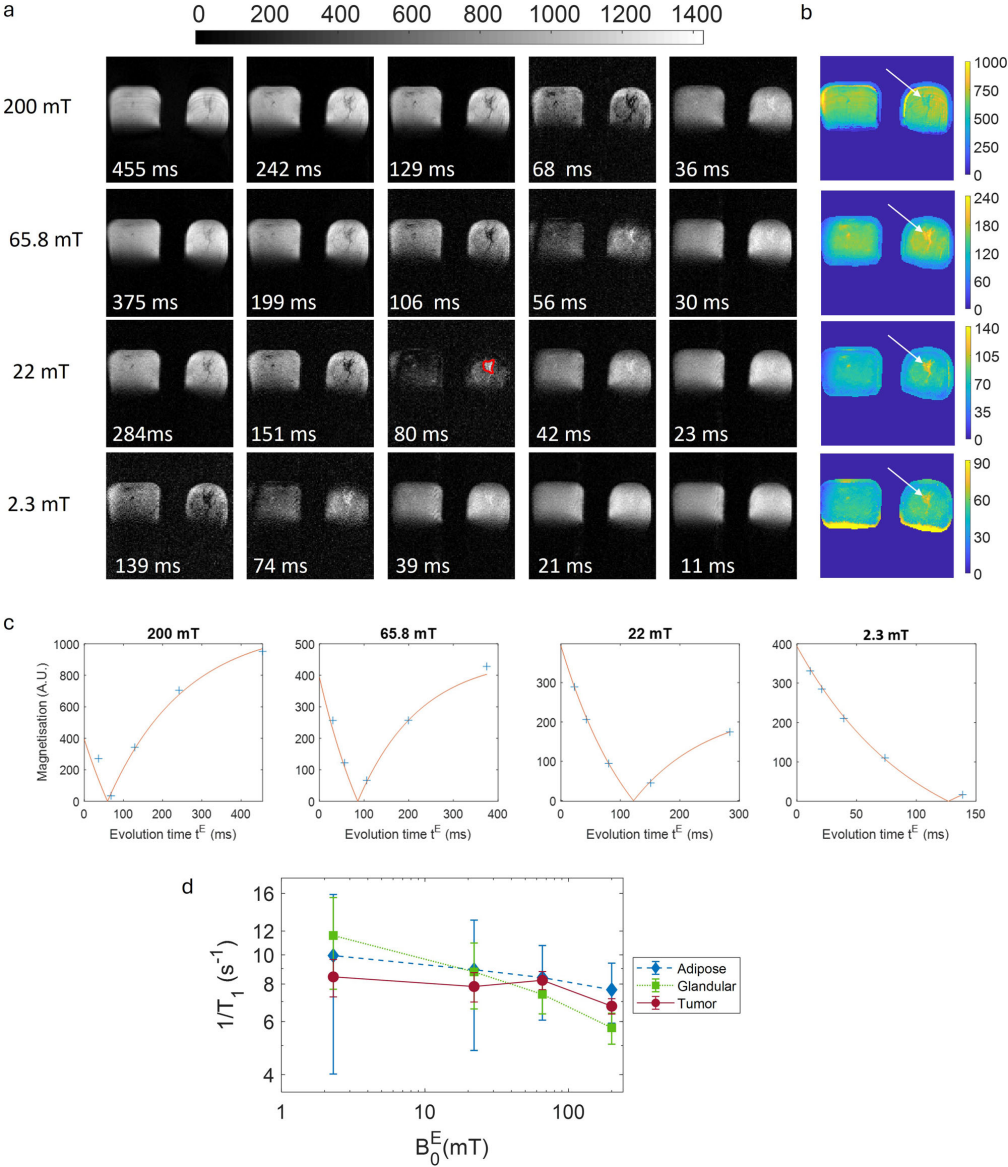
Typical images and data obtained from the FCI scans. **a** Typical axial images from volunteer P2 showing the quality of our FCI prototype at the four B_0_^E^ fields. Inside each image, the evolution time t^E^ is given in ms. The grey colour bars show signal magnetisation intensities in arbitrary unit (A.U). Volunteer P2 has a large breast that appear slightly square due to the restricted RF coil size. **b** T_1_ maps at B_0_^E^ = 2.3, 22, 65.8 and 200 mT. Heterogeneous T_1_ distribution throughout the breast is observed. The arrow shows the regions corresponding to the tumours (elevated values in yellow). The colour bars show the T_1_ values in ms. **c** Magnetisation relaxation recovery of tumour ROIs fitted using a monoexponential model in DCIS. The tumour ROI (in red colour) is depicted in image at B_0_^E^ = 22 mT, t^E^ = 80 ms. **d** 1/T_1_-NMRD profiles from homogeneous ROIs of the DCIS, glandular and adipose tissues for this dataset (*n* = 1). The error bars are the fitting errors which were set equal to 1 sigma, the raw data is available in Supplementary Table 1.

The total scan duration was limited to 60 min for comfort, including calibrations, axial and sagittal navigator images and FCI images. We designed an FCI examination with five evolution times per field strength (Fig. 3a), which was a good compromise between speed and accuracy for T_1_ estimation. This left us with enough time to explore four evolution fields in 30 min. We selected 200, 65.8, 22, and 2.3 mT due to the different relaxation behaviours in breast tissues above and below 22 mT, and to assess quadrupolar relaxation at 65.8 mT, based on our previous experiments with excised breast tissues (see Fig. S1).

### Data analysis

The raw FCI images were processed using MATLAB 2019a (The MathWorks, Inc., Natick, Massachusetts, United States) to remove artefacts^33^ and by tailor-made TGV-based software written in Python to obtain T_1_ maps^34^. Three Regions-of-Interests (ROIs) were manually drawn on the FCI scans of contralateral breasts to select adipose and glandular tissue and on abnormal breast to select the lesion. ROI placements were blind to histology data but were made with reference to the lesions observed from conventional imaging. P7 had a partial mastectomy before recruitment, therefore the ROI in healthy adipose tissue was selected from the abnormal breast while glandular tissue was not clearly seen. This dataset was kept in the study since radiotherapy targeted the previous diseased breast, and the baseline normal measurements matched normal tissues in the bilateral breast cases.

The ROIs were used to average the magnetisation from the FCI images and to obtain the 1/T_1_ NMRD profiles from curve fitting using a mono-exponential model:

1 $${{{\rm{S}}}}({{{{{\rm{B}}}}}_{0}}^{{{{\rm{E}}}}},{{{{\rm{t}}}}}^{{{{\rm{E}}}}})={{{\rm{A}}}}\left[({{{{{\rm{M}}}}}_{0}}^{{{{\rm{P}}}}}{{{\rm{\hbox{--}}}}}{{{{{\rm{M}}}}}_{0}}^{{{{\rm{E}}}}})\exp (-{{{{\rm{t}}}}}^{{{{\rm{E}}}}}/{{{{\rm{T}}}}}_{1})+{{{{{\rm{M}}}}}_{0}}^{{{{\rm{E}}}}}\right]$$

where A is a scaling factor that accounts for proton density, magnetic susceptibility, and RF chain efficiency, M_0_^P^ is the longitudinal magnetisation obtained after the polarisation stage, M_0_^E^ is the equilibrium magnetisation level during the evolution stage and t^E^ is the evolution time. In conventional MRI, this equation is simpler as there is a direct relationship between M_0_^P^ and M_0_^E^, but for FCI the model has to take into account the change in equilibrium magnetisation M_0_^E^, which depends on the evolution field.

The NMRD profiles of the different tissues were then fitted to derive the dispersion (β parameter) at fields below (β parameter at low fields, β_L_) and above (β parameter at high fields, β_H_) 22 mT using a power law model:

2 $$1/{{{{\rm{T}}}}}_{1}({{{{{\rm{B}}}}}_{0}}^{{{{\rm{E}}}}})={{{\rm{\alpha }}}}{{{{{\rm{B}}}}}_{0}}^{{{{\rm{E}}}}\left(-{{{\rm{\beta }}}}\right)}$$

where B_0_^E^ is the evolution field, and α and β are the amplitude and dispersion of the power law model respectively. In a log-log plot, α corresponds to the value of the NMRD profile at 1 mT and β to the slope of the curve. This model was chosen for its simplicity, which allows extracting structural information from a limited number of points in the T_1_ NMRD profiles.

The amplitude of the QPs peaks at 65.8 mT was estimated by subtracting the baseline provided from interpolation.

The Signal-to-Noise Ratio (SNR) was measured at each field on the magnitude FCI image acquired at the longest evolution time, with one ROI positioned on the contralateral healthy breast and the other on the image background, using the Rayleigh correction factor^35^ as

3 $${{{\rm{SNR}}}}=0.66{{{\rm{xS}}}}({{{{\rm{ROI}}}}}_{{{{\rm{S}}}}})/{{{\rm{S}}}}({{{{\rm{ROI}}}}}_{{{{\rm{N}}}}})$$

where S(ROI_S_) is the mean intensity from the ROI on the contralateral healthy tissue and S(ROI_N_) is the mean intensity from the ROI on background noise.

The Contrast-to-Noise Ratio (CNR) was measured from the same FCI images using the ROIs from tumour and adjacent healthy tissue as

4 $${{{\rm{CNR}}}}=\frac{\left|S({{{\rm{ROI}}}}_{T})-S({{{\rm{ROI}}}}_{H})\right|}{S({{{\rm{ROI}}}}_{N})\times 0.66}$$

where S(ROI_T_) is the mean signal intensities from the ROIs in tumour and S(ROI_H_) is the mean signal intensities from the ROIs in adjacent healthy tissue which generally includes a mix of adipose and glandular tissue.

The differences in T_1_ between tumour and adjacent healthy tissue, referred to as tumour-to-background contrast ratio, were calculated as the differences from the corresponding ROIs in T_1_ maps and are provided in percent of average T_1_, using the equation

5 $$\Delta {{{{\rm{T}}}}}_{1} \% =\frac{{T}_{1}({{{\rm{ROI}}}}_{T})-{T}_{1}({{{\rm{ROI}}}}_{H})}{[{T}_{1}\left({{{\rm{ROI}}}}_{T}\right)+{T}_{1}({{{\rm{ROI}}}}_{H})]/2}\times 100$$

where ${T}_{1}({{ROI}}_{T})$ and ${T}_{1}({{ROI}}_{H})$ are the mean T_1_ values from the ROIs in tumour and adjacent healthy tissue respectively from T_1_ maps. All these methodological specifications are reported in Table 2.

**Table 2:** SNR and CNR from magnitude FCI images at the longest evolution time, and %ΔT_1_ from T_1_ maps, calculated between tumours and adjacent uninvolved breast tissue at different field strengths B_0_^E^ (*n* = 9)

B_0_^E^	200 mT		65.8 mT		22 mT		2.3 mT	
	Median	Range^a^	Median	Range^a^	Median	Range^a^	Median	Range^a^
SNR	58	27–101	29	12–58	16	3.5–26	4.8	2.4–9.2
CNR	29	8.4–67	11	3.5–38	10	3.5–18	3.3	0.39–5.2
%ΔΤ_1_	31	−14–65	32	10–89	62	25–116	48	23–109

^a^The values are presented as median, minimum, and maximum values across all volunteers.

### Statistics and reproducibility

Each volunteer was scanned once only due to management constraints and limitation from long scan time. The sample size was chosen to provide a first estimate of the confidence intervals of the potential biomarkers, to plan for future studies^36^.

Statistical analyses were performed using SPSS Statistics 28 (IBM corp., Chicago, United States). FCI biomarkers (1/T_1_, β parameter, QP peak amplitude) comparisons between volunteers with invasive and volunteers with non-invasive tumours were made using Mann–Whitney U tests. FCI biomarkers (1/T_1_, β_L_, β_H_, QP peak amplitude) comparisons within each volunteer between tumours and normal tissues were performed using Wilcoxon signed-rank tests. Tests were two-sided and a *P* value below 0.05 was considered statistically significant.

Data are given as median values with interquartile range in parenthesis. Values were visualised using boxplots; on each box, the central line indicates the median, and the bottom and top edges of the box indicate the 25th and 75th percentiles, respectively. The whiskers, bottom and top indicate the minimum and maximum, respectively.

## Results

### FCI images

Typical FCI data are shown in Fig. 3 (volunteer P2), including FCI images and T_1_ maps for the four magnetic fields we have used (Fig. 3a, b, respectively). Magnetisation relaxations recovery of the tumour region are shown in Fig. 3c and exhibit a monoexponential behaviour for the four fields, but we cannot exclude a biexponential behaviour if using a finer evolution time sampling for smaller values of t^E^, as reported in other work^11^. Figure 3d shows the 1/T_1_-NMRD profiles extracted from images of P2 over homogeneous ROIs of the DCIS, glandular and adipose tissues, with the largest T_1_ variations observed in breast tissues (86 to 175 ms). Qualitative differences appear at first sight in the dispersion profiles, which can be measured quantitatively on the T_1_ values between tumour tissues and normal breast.

**Fig. 4:**
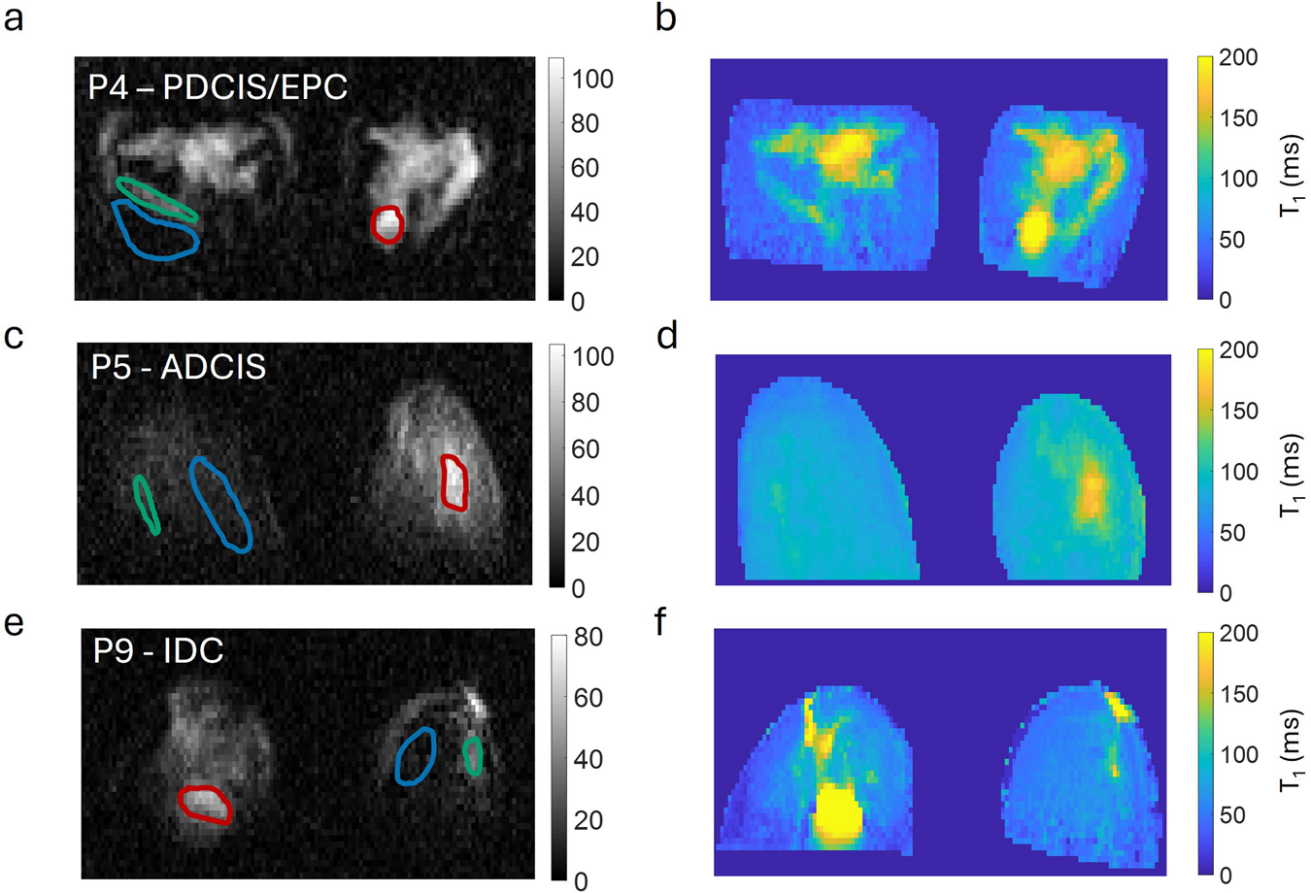
Typical FCI tissue contrast in magnitude and T_1_ maps. FCI images at 22 mT and t^E^ of 88 ms for (**a**). PDCIS/EPC (papillary ductal carcinoma in situ/ Encysted papillary carcinoma, P4), (**c**) ADCIS (apocrine ductal carcinoma in situ, P5), and (**e**). IDC (Invasive Ductal Carcinoma, P9). The lesions are drawn in red and appeared clearly hyperintense. Adipose and glandular tissues are drawn in blue and green respectively. **b**, **d**, **f** The corresponding T_1_ maps for volunteers P4, P5, and P9. The colour bar shows the T_1_ in ms.

For all volunteers, the tumour region is easily visible (see SNR and CNR values in Table 2) and the tumour-to-background contrast ratio (%ΔT_1_) is more pronounced at lower fields (median [range]%: 31 [−14–65]% at 200 mT and 48 [23–109]% at 2.3 mT, with maximum value at 22 mT: 62 [25–116]%). At this 22 mT, the corresponding FCI images and T_1_ maps clearly discriminate tumours from glandular and from adipose tissues, as shown in Fig. 4 for different tumour types.

### Comparison of FCI and conventional imaging

**Fig. 5:**
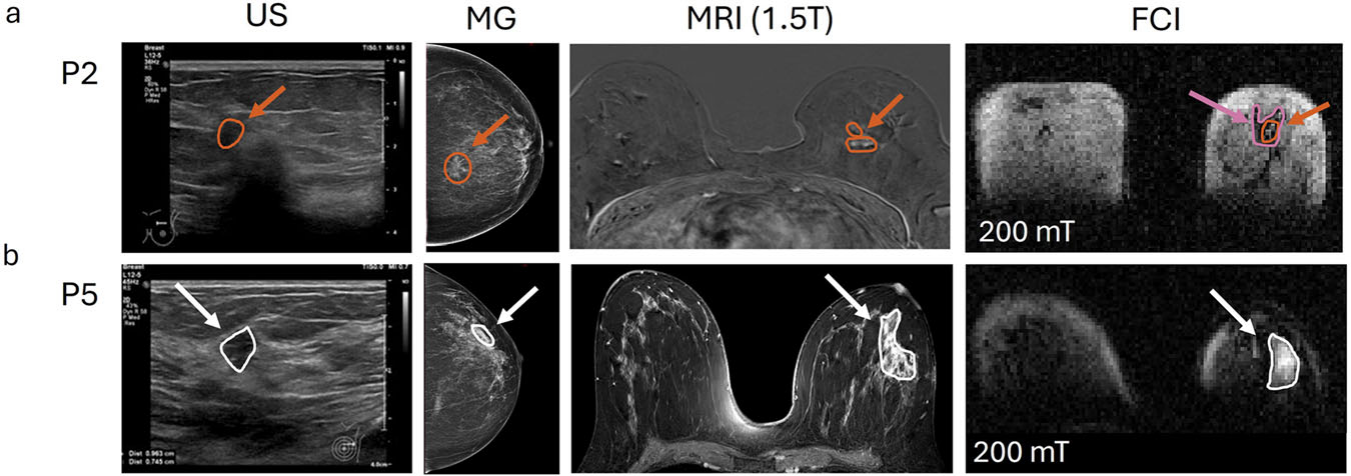
Comparison of images from US, MG, MRI and FCI. Ultrasound (US), Mammogram (MG), axial MRI and FCI images from volunteers P2 and P5. The ROIs highlight areas that correspond to the tumour. **a** P2 presented two distinct tumours in the left breast: ILC surrounded by DCIS. The ILC is detected by US and MG and is shown as bright areas in MRI (orange ROIs indicated by orange arrows). The DCIS is not seen in US, MG, and MRI. FCI detected both lesions with bright area corresponding to ILC and dark area corresponding to DCIS as indicated by orange and purple ROIs with arrows respectively in FCI images (breast was squeezed due to the restricted RF coil size). **b** For volunteer P5, the tumour is localised at the left breast, and it is indicated by white ROIs and arrows; For P5, the DCIS was not seen in MG but was retrospectively analysed to show a lesion of 25 mm. US showed a smaller mass of 11 mm. FCI detected the DCIS area (tumour in white ROI indicated by white arrows). (MG: Mammography, US: Ultrasounds, FCI: Field-Cycling Imaging, ILC: Invasive Lobular Carcinoma, DCIS: Ductal Carcinoma In Situ, ROI: Region Of Interest).

FCI successfully identified the tumour in all 10 cases, including the two cases (P1 and P4) where volunteers had dense breast parenchyma (BI-RADS density category C) (Fig. 4a). However, since this study was not blinded, further investigation with a blinded design is required to validate this finding. For the other imaging modalities, 3 out of 8 DCIS cases (38%) were not detected by US and/or MG (P1, P2, P7) and one DCIS case (13%) was not detected by MRI (P2). One DCIS case was retrospectively detected by MG (P5). Typical images (US, MG, MRI and FCI) from P2 and P5 are presented in Fig. 5, showing the regions corresponding to the tumour (as indicated by ROIs and arrows). For P2, US, MG, and MRI detected only the invasive core (ILC, ROIs in orange), not showing the surrounding DCIS area. FCI detected both the invasive core (hyperintense area indicated by orange ROI and arrow) and the surrounding DCIS area (hypointense area indicated by purple ROI and arrow), with different contrast levels (Fig. 5a). For P5, DCIS was only detected retrospectively in MG while FCI clearly detected the DCIS area (as indicated by ROIs and arrows in white) (Fig. 5b).

### FCI NMRD profiles and biomarkers of breast cancer detection

**Fig. 6:**
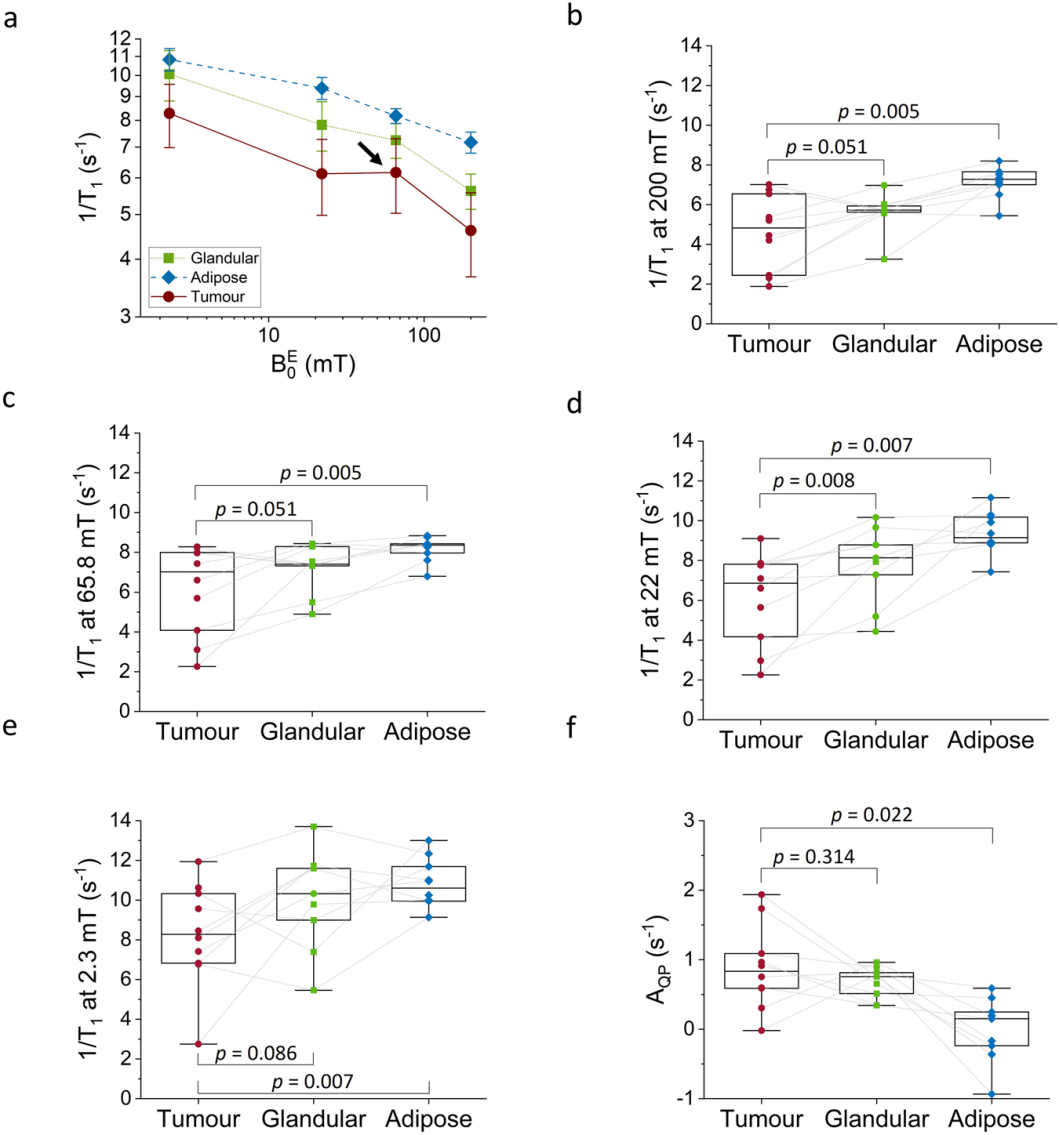
Quantitative analysis of the FCI dispersion profiles for tumour discrimination. **a** Average 1/T_1_-NMRD profiles for all the data, obtained from the three ROIs shown in Fig. 4: adipose tissues in blue diamond (*n* = 9), glandular tissues in green square (*n* = 8) and tumour lesions in red circle (*n* = 10). Clear trends can be observed between healthy and tumour tissues, despite the inter-volunteer variability. The error bar shows the standard deviation across volunteers and are set to +/- sigma. The amplitude of QP peaks (arrow at data point at 65.8 mT) is clearly increased in tumours. We have all individual points in box plots in Fig. 6b–e, therefore Fig. 6a is presented as mean with error bars for clarity since it only serves as an illustration. **b**–**f** The 1/T_1_ values at 200, 22, 2.3 mT and the amplitude of the QP peak are shown in box plots. Box plots show the median and 25th and 75th percentiles and the whiskers shows the maximum and minimum. *P* values to test differences in the 1/T_1_ values between tumour and healthy tissues either glandular (*n* = 8) or adipose (*n* = 9) were computed using a two-sided Wilcoxon signed-rank test. The raw data for all figures is available in Supplementary Tables 1 and 3.

Figure 6a shows the corresponding 1/T_1_-NMRD profiles of three ROIs selected: in the tumour, in non-involved adipose tissues, and in non-involved glandular tissues. All the NMRD curves exhibit a general 1/T_1_ decrease with the magnetic field strength, with a variation of a factor 2 to 5 between 200 and 2.3 mT depending on the tissue types. More interestingly, 1/T_1_ values from tumours are significantly lower than adipose tissues at all fields, (or conversely T_1_ is longer) (Fig. 6b–e). The 1/T_1_ values from tumours are significantly lower than glandular tissues at 22 mT (6.8 [3.9–7.8] s^−1^ vs 8.1 (6.2–9.2) s^−1^, two-sided Wilcoxon signed-rank test, Z = −2.7, *P* = 0.008, effect size = 0.854). Relaxation rate enhancements at 65.8 mT, which results from the ^14^N−^1^H quadrupolar coupling (arrow indicated on Fig. 6a), appear larger in tumours (0.83 [0.52–1.2] s^−1^) compared with glandular tissues (0.76 [0.43–0.85] s^-1^, Fig. 6f). Note that no QP signal is detected in adipose tissue, as expected from tissues with low protein content. This also agrees with ex vivo experiments^21^, indicating that QP relaxation can be used to improve breast tumour detection. The model parameters extracted from the data are summarised in Table 3 with their significance levels, including QP relaxation enhancements, showing that 1/T_1_ and A_QP_ are candidate biomarkers of molecular dynamics for the detection of breast cancer against breast tissues.

**Table 3:** 1/T_1_ values and parameters derived from the power law mathematical model: β parameter at high-field (β_H_) and low-field (β_L_) and QP peak amplitudes (A_QP_)

	Tumour (*n* = 10)^a^	Glandular tissue (*n* = 8)^a^	Adipose tissue (*n* = 9)^a^	Tumour vs glandular			Tumour vs adipose		
				Z	*P*	Effect size	Z	*P*	Effect size
1/T_1, 200mT_ (s^−1^)	4.8 (2.4–6.6)	5.7 (5.6–6.0)	7.3 (6.9–7.7)	−2.0	0.051	0.632	−2.8	0.005	0.885
1/T_1, 65.8mT_ (s^−1^)	7.0 (3.8–8.0)	7.4 (6.4–8.3)	8.4 (7.9–8.5)	−2.0	0.051	0.632	−2.8	0.005	0.885
1/T_1, 22mT_ (s^−1^)	6.8 (3.9–7.8)	8.1 (6.2–9.2)	9.1 (8.9–10.2)	−2.7	0.008	0.854	−2.7	0.007	0.854
1/T_1, 2.3mT_ (s^−1^)	8.3 (6.8–10.4)	10.3 (8.2–11.7)	10.6 (9.9–11.9)	−1.7	0.086	0.538	−2.7	0.007	0.854
β_H_	0.14 (0.04–0.21)	0.19 (0.08–0.21)	0.12 (0.08–0.16)	−0.89	0.374	0.281	−0.56	0.575	0.177
β_L_	0.10 (0.03–0.19)	0.10 (0.09–0.13)	0.06 (0.05–0.07)	−0.77	0.441	0.243	−1.8	0.074	0.569
A_QP_ (s^−1^)	0.83 (0.52–1.2)	0.76 (0.43–0.85)	0.15 (−0.27–0.30)	−1.0	0.314	0.316	−2.3	0.022	0.727

^a^All data are given as median and interquartile range in parenthesis. The *P* values were derived by two-sided Wilcoxon signed rank tests between tumour and glandular tissues and between tumour and adipose tissues.

### FCI biomarkers of breast cancer invasion

**Fig. 7:**
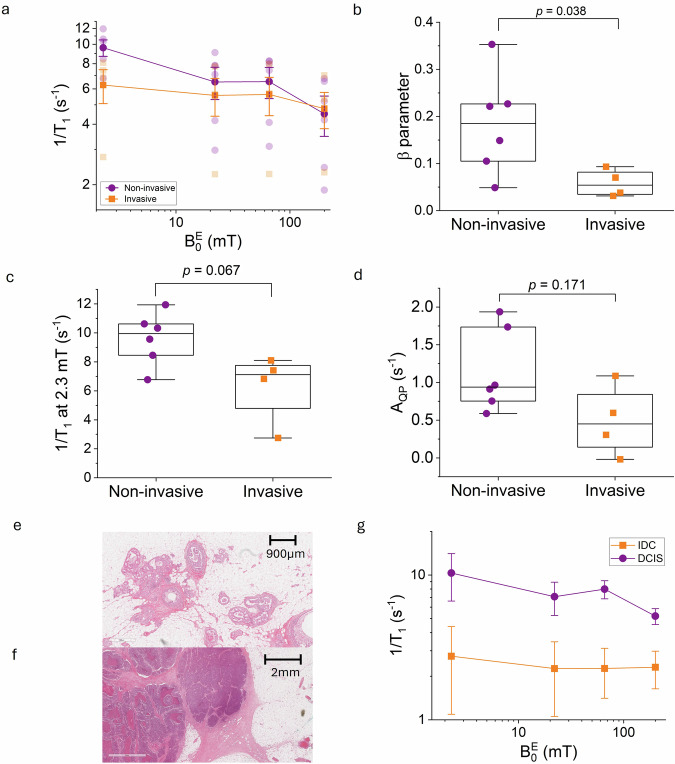
Quantitative analysis of the FCI dispersion profiles for tumour characterisation. **a** Tumour dispersion profiles of invasive tumours (P2, P3, P6, P9; orange square) and non-invasive tumours (P1, P2, P4, P5, P7, P8; purple circle, *n* = 6), showing different behaviours of NMRD profiles. The error bar shows the standard deviation across groups and are set to 1 sigma. Plots **b**, **c**, **d** and **g** present other views of the same dataset, with *n* = 4 and 6 for invasive and non-invasive tumours respectively: (**b**) The dispersion (β parameter), (**c**) relaxation rate at 2.3 mT, and (**d**) QP peak amplitude between invasive and non-invasive tumours are shown in box plots. **e**, **f** Representative HE histology images of non-invasive (DCIS, P8) and invasive (IDC, P10) breast tumour respectively. **g** Dispersion profiles of non-invasive (DCIS, P8, purple circle) and) invasive (IDC, P9, orange square) tumour. Box plots show the median and 25th and 75th percentiles and the whiskers shows the maximum and minimum. *P* values to test differences in the dispersion (β parameter), relaxation rate at 2.3 mT, and QP peak amplitude between invasive and non-invasive tumours were computed using a two-sided Mann–Whitney U test. (FCI: Field-Cycling Imaging, NMRD: NMR Dispersion, DCIS: Ductal Carcinoma In Situ, IDC: Invasive Ductal Carcinoma, QP: Quadrupolar Peaks, HE: Hematoxylin and Eosin). The raw data for all figures are available in Supplementary Tables 2 and 3.

The 1/T_1_ NMRD profiles from breast tumours show some variability between volunteers, and further categorisation of these profiles according to tumour invasiveness show significant differences between non-invasive and invasive tumours (Fig. 7a). These appear in the dispersion parameter β (0.19 [0.09–0.26] in non-invasive vs 0.05 [0.03–0.09] in invasive tumours, two-sided Mann–Whitney U test, Z = −2.1, *P* = 0.038, effect size = 0.674, Fig. 7b). The 1/T_1_ relaxation rate at 2.3 mT is markedly higher in non-invasive tumours, although not significant (9.9 [8.0–10.9] s^−1^ in non-invasive against 7.1 [3.8–7.9] s^−1^ in invasive, two-sided Mann–Whitney U test, Z = −1.9, *P* = 0.067, effect size = 0.607, Fig. 7c). These differences in tissue relaxation time T_1_ also appear clearly within volunteer P2, where the tumour core appears brighter (high invasion phenotype, higher T_1_) and is surrounded by the darker DCIS (non-invasive region, lower T_1_), from 2.3 mT to 200 mT (see Fig. 5a). These agree with the literature^11,37^, supporting the claim that T_1_ relaxation at ultra-low fields in living tissues is a relevant biomarker discriminating invasion from non-invasion. This phenomenon is linked to the transmembrane water exchange, which has been found to be more rapid in tissues with glioma cells invasion^12,38^. Also, QP peak amplitudes are markedly higher for non-invasive tumours, although not significant (0.9 [0.7–1.8] s^−1^ in non-invasive vs 0.5 [0.06–1.0] s^−1^ in invasive, two-sided Mann–Whitney U test, Z = −1.5, *P* = 0.171, effect size = 0.472, Fig. 7d). One can also note that the trend followed by the tumour dispersion profiles below 2.3 mT suggests that larger tissue contrast can be observed at lower fields, potentially providing even greater discrimination between tissues, with more powerful tests for the detection of breast tumour invasiveness.

HE Histology confirmed if tumours were invasive or not. Figure 7e, f shows typical HE histology images for non-invasive DCIS (P8) and invasive IDC (P10) respectively. 1/T_1_ NMRD profiles from these two cases are shown in Fig. 7g depicting the different dispersions (β of 0.15 in non-invasive vs 0.04 in invasive), which supports the role of molecular dynamics as relevant biomarkers to discriminate tumour invasion from non-invasion.

## Discussion

This study is a first step in determining the potential of FCI and its unique biomarkers related to breast cancer pathology. To the best of our knowledge, we have shown for the first time that NMRD profiles can be extracted from FCI images in vivo from people living with breast cancer, providing clinically relevant information about tumour presence and invasiveness without the need for contrast agents.

The image quality of our FCI prototype is sufficient to generate accurate T_1_ maps and NMRD profiles. The tissue contrast successfully discriminates between tumours, adipose and glandular tissues, and its dispersion discriminates between invasive and non-invasive tumours, including IDC, ILC, and DCIS, resulting from the changes in the molecular dynamics of water. The QP peak amplitude is slightly lower in invasive tumours compared with non-invasive ones, although the dispersion of the 1/T_1_ – NMRD profiles is a more significant discriminator. Consequently, QP relaxation may not be a biomarker of breast cancer in isolation but may be combined with the other FCI signals to provide more accurate measurement of tumour invasiveness and spread, with applications in efficient surgery guiding and potential impact in treatment planning.

FCI detects the tumour area across a variety of breast density categories (from A to C). Indeed, the dispersion levels are markedly different between glandular and tumour tissues. However, due to the small sample size of nine volunteers, further exploration is needed to assess the potential of FCI to detect breast cancer irrespectively of breast density. If confirmed, this could be a major asset in dense breasts, especially when scanning young persons.

The mixed lesion from P2 is markedly under-estimated by US, MG, and MRI as confirmed from histology, yet FCI can detect the two regions separately. This illustrates an important issue with breast imaging, where some types of breast cancers remain difficult to detect and under-estimations may lead to repeated operations due to involved margins for breast conserving surgery, while overestimation may lead to unnecessary mastectomies. This includes missing ductal carcinomas in situ or lobular cancer due to its discohesive and infiltrative nature, subtle calcifications, and breast density which both lead to biases in tumours diagnosis. From this study, it appears that FCI can overcome some of these limitations and have a complementary role in the detection of breast cancers.

Additionally, the results obtained agree with preclinical FFC-NMR studies of cancer mouse models ex vivo and in vivo, which showed a causal relationship between the T_1_ increase (or 1/T_1_ decrease) in tumours at low fields (typically below 50 mT) and the increase of transmembrane water exchanges driven by the increased cell metabolism in invasive tumours^12,14,38^.

The prototype scanner used in this study is the first ever to be used for clinical studies and has therefore several important limitations. The FCI magnet homogeneity is poor and small lesions have been missed because the single slice multi-field acquisition was not correctly positioned due to poor contrast in the multi-slice anatomical navigator images at 0.2 T. Moreover, the single-slice scans and limited SNR and CNR made it challenging to detect the whole tumour, but our results clearly demonstrate the potential that this technology has in breast cancer. The narrow bore (50 cm diameter) also made recruitment challenging, and several technical issues arose from software incompatibilities. FCI technology was also being developed in parallel with the study, which led to unexpected down times when repairs had to be made on the RF and magnet amplifier systems. Yet, FCI scans are quiet and relatively comfortable and seemed well tolerated.

A faster, wider-bore FCI system is currently under construction in a clinical setting to start a larger study which will address these limitations, bringing this technology closer to the clinic. While this study focuses on breast cancer, other on-going works show potential B_0_-dependent biomarkers appear across other pathologies, particularly in cancers. The potential for imaging without contrast agents is also a great benefit as it simplifies the examination and eliminates the need for Gadolinium-based contrast agents where there is concern about Gadolinium deposition^29^.

More broadly, FCI can inform on fixed-field MRI measurements at set magnetic field strengths selected to highlight the best contrast levels for a specific disease or pathophysiological process. For instance, our results suggest that the contrast between tumours and healthy tissues is better perceived at 22 mT, while contrast between invasive and non-invasive regions are better targeted at 2.3 mT (Fig. 7g).

To conclude, this is the first application of in vivo FCI in persons living with breast cancer to the best of our knowledge. FCI shows high potential for breast tumour characterisation as it provides new biomarkers for tumour presence and invasiveness. FCI may complement diagnosis of current imaging modalities by offering new contrast sources that inform on pathophysiological processes in breast cancer. This may improve breast cancer diagnosis since the detection of invasive phenotypes remain challenging by current medical imaging modalities.

## Supplementary information

Supplementary Information

## Data Availability

Source data for Figs. 6 and 7a–d, g can be found in Supplementary Tables 1–3, which contains the results from our previous experiments on excised breast tissues which support the selection of our scanning protocol. All other data that support the findings of this study are available on request from the corresponding author (L.M.B).

## Abbreviations

**FFC-NMR**: Fast Field-Cycling Nuclear Magnetic Resonance
**FCI**: Field Cycling Imaging
**NMRD**: Nuclear Magnetic Resonance Dispersion
**QP**: Quadrupolar
**RF**: RadioFrequency
**SNR**: Signal to Noise Ratio
**CNR**: Contrast to Noise Ratio
**DCIS**: Ductal Carcinoma in Situ
**ILC**: Invasive Lobular Carcinoma
**HE**: Hematoxylin and Eosin
**US**: UltraSound
**MG**: MammoGraphy
**NAD**: No Abnormality Detected

## Code availability

The TGV software developed to analyse the FCI images is available under the Apache license v2.0 (https://github.com/IMTtugraz/PyQMRI). The details of TGV software are described in reference^34^. The code for the pre-processing of the FCI images is available at the following location^39^: https://github.com/JRossFFC/FFC_Processing.
