# Supplementary material for: Field cycling imaging to characterise breast cancer at low and ultra-low magnetic fields below 0.2 T
Source: Commun Med (Lond). 2024 Oct 30;4:221. doi: 10.1038/s43856-024-00644-2 (PMC11526037; doi:10.1038/s43856-024-00644-2)
Supplement: Supplementary file 1 — Supplementary Information [file 43856_2024_644_MOESM1_ESM.pdf]

Field Cycling Imaging to characterise breast cancer at low and ultra-low magnetic fields below 0.2T

Authors

Vasiliki Mallikourti[1], P. James Ross[1], Oliver Maier[2], Katie Hanna[3], Ehab Husain[4], Gareth R. Davies[1], David J. Lurie[1], Gerald Lip[4], Hana Lahrech[5], †Yazan Masannat[4,6], †Lionel M. Broche[1]*

*Corresponding author: Lionel Broche:

email: l.broche@abdn.ac.uk

Affiliations

[1]Aberdeen Biomedical Imaging Centre, University of Aberdeen, Aberdeen, United Kingdom

[2]Institute of Biomedical Imaging, Graz University of Technology, Graz, Austria

[3]Institute of Medical Sciences, University of Aberdeen, Aberdeen, United Kingdom

[4]Breast Unit, Aberdeen Royal Infirmary, Aberdeen, United Kingdom

[5]University Grenoble Alpes, Inserm U1205, BrainTech Lab, Grenoble, France

[6]School of Medicine, Medical Sciences and Nutrition, University of Aberdeen, Aberdeen, United Kingdom

†: These two authors contributed equally to this article

Supplementary Methods

A total of 10 female breast cancer patients underwent mastectomies at the Aberdeen Royal Infirmary, Scotland. Each mastectomy was cut at three sites 24h after fixation in formaldehyde: within the tumour, adjacent to the tumour and at the resection margin providing a total of 30 excised breast tissues. 1/T1 dispersion curves were acquired from each tissue sample using the benchtop relaxometer (SMARtracer; Stelar S.r.l., Mede, Italy). Each sample was scanned at 60 different evolution fields in the 0.001-8 MHz proton Larmor frequency range. The patient diagnostic was confirmed from pathology reports.

Supplementary Results

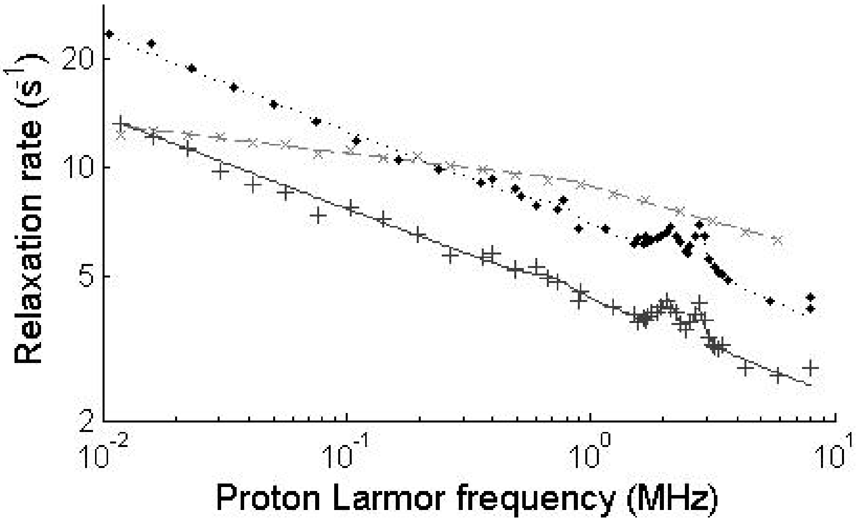

Figure S1: Dispersion curves from breast samples taken within a lobular carcinoma (dots), at the resection margin (light crosses), and adjacent to the tumour (dark crosses), after fixation following the methods described in Supplementary Methods. Large variations can be seen in the shape of the dispersion curve even though the samples of breast tissues presented similar appearance under the microscope. In particular, we can see a change in the dispersion of the tissue margin sample at a frequency of 1 MHz, or equivalently 22 mT. Quadrupolar peaks were also observed in tumour and peritumoral regions, and appear as peaks between 1.5 and 3.5 MHz.

Supplementary Table 1: R_1 values found from the FCI images of the volunteers, in s^{-1}.

P.	Invasiveness	Glandular				Adipose				Tumour			
		**200 mT**	**65.8 mT**	**22 mT**	**2.3 mT**	**200 mT**	**65.8 mT**	**22 mT**	**2.3 mT**	**200 mT**	**65.8 mT**	**22 mT**	**2.3 mT**
1	NI	5.568	5.500	4.444	5.460	5.445	6.785	7.422	9.136	2.441	4.084	4.175	6.769
2	I	5.723	7.395	8.780	11.60	7.658	8.405	8.929	9.947	7.011	7.960	7.751	8.099
2	NI	5.723	7.395	8.780	11.60	7.658	8.405	8.929	9.947	6.755	8.233	7.850	8.452
3	I	5.621	8.282	9.656	11.73	6.503	7.960	9.355	11.01	4.443	5.700	6.612	6.826
4	NI	3.256	4.901	5.192	8.992	7.206	7.595	10.18	11.69	1.883	3.106	2.972	9.565
5	NI	6.023	8.428	10.16	13.70	7.087	8.262	10.29	12.34	4.204	7.423	7.809	11.94
6	I	6.973	8.410	8.131	10.32	8.197	8.828	9.921	10.97	5.364	6.589	5.650	7.411
7	NI	-	-	-	-	7.345	8.306	8.881	10.25	6.543	8.272	9.096	10.62
8	NI	5.934	7.309	7.272	7.394	7.532	8.761	11.16	13.00	5.203	7.987	7.086	10.33
9	I	5.782	7.534	7.924	9.787	7.003	8.423	8.811	9.993	2.306	2.263	2.258	2.749
Average		5.610	7.220	7.695	9.873	7.108	8.147	9.438	10.93	4.615	6.162	6.126	8.276
Standard deviation		1.050	1.335	2.013	2.610	0.781	0.634	1.089	1.244	1.905	2.264	2.294	2.593

Supplementary Table 2: Dispersion β of the R_1 NMRD profiles measured from the FCI scans

Patient	Invasiveness	Glandular		Adipose		Tumour		
		β_H	β_L	β_H	β_L	β_H	β_L	$\beta_{Average}$
1	NI	-0.0979	0.0894	0.1346	0.0902	0.2332	0.2098	0.2215
2	I	0.1858	0.1209	0.0667	0.0469	0.0436	0.0191	0.0313
2	NI	0.1858	0.1209	0.0667	0.0469	0.0652	0.0321	0.0487
3	I	0.2350	0.0845	0.1579	0.0706	0.1726	0.0139	0.0932
4	NI	0.2027	0.2386	0.1500	0.0602	0.1982	0.5076	0.3529
5	NI	0.2271	0.1298	0.1617	0.0790	0.2689	0.1843	0.2266
6	I	0.0667	0.1037	0.0829	0.0435	0.0225	0.1179	0.0702
7	NI	-	-	0.0824	0.0622	0.1431	0.0673	0.1052
8	NI	0.0883	0.0072	0.1706	0.0665	0.1341	0.1636	0.1489
9	I	0.1369	0.0917	0.0997	0.0547	-0.0093	0.0855	0.0381

Supplementary Table 3: Amplitude of the quadrupolar peaks measured from the FCI scans

Patient	Invasiveness	Quadrupolar peak amplitude (s^{-1})		
		**Glandular**	**Adipose**	**Tumour**
1	NI	0.512	0.451	0.912
2	I	0.342	0.150	0.597
2	NI	0.342	0.150	0.964
3	I	0.961	0.193	0.305
4	NI	0.813	-0.934	0.753
5	NI	0.654	-0.238	1.736
6	I	0.895	-0.169	1.088
7	NI		0.248	0.587
8	NI	0.756	-0.363	1.937
9	I	0.791	0.589	-0.020
